# Metformin Downregulates the Insulin/IGF-I Signaling Pathway and Inhibits Different Uterine Serous Carcinoma (USC) Cells Proliferation and Migration in p53-Dependent or -Independent Manners

**DOI:** 10.1371/journal.pone.0061537

**Published:** 2013-04-19

**Authors:** Rive Sarfstein, Yael Friedman, Zohar Attias-Geva, Ami Fishman, Ilan Bruchim, Haim Werner

**Affiliations:** 1 Department of Human Molecular Genetics and Biochemistry, Sackler School of Medicine, Tel Aviv University, Tel Aviv, Israel; 2 Gynecologic Oncology Unit, Department of Obstetrics and Gynecology, Meir Medical Center, Kfar Sava, Israel; Universidad Miguel Hernández de Elche, Spain

## Abstract

Accumulating epidemiological evidence shows that obesity is associated with an increased risk of several types of adult cancers, including endometrial cancer. Chronic hyperinsulinemia, a typical hallmark of diabetes, is one of the leading factors responsible for the obesity-cancer connection. Numerous cellular and circulating factors are involved in the biochemical chain of events leading from hyperinsulinemia and insulin resistance to increased cancer risk and, eventually, tumor development. Metformin is an oral anti-diabetic drug of the biguanide family used for treatment of type 2 diabetes. Recently, metformin was shown to exhibit anti-proliferative effects in ovarian and Type I endometrial cancer, although the mechanisms responsible for this non-classical metformin action remain unclear. The insulin-like growth factors (IGFs) play a prominent role in cancer biology and their mechanisms of action are tightly interconnected with the insulin signaling pathways. Given the cross-talk between the insulin and IGF signaling pathways, the aim of this study was to examine the hypothesis that the anti-proliferative actions of metformin in uterine serous carcinoma (USC) are potentially mediated *via* suppression of the IGF-I receptor (IGF-IR) pathway. Our results show that metformin interacts with the IGF pathway, and induces apoptosis and inhibition of proliferation and migration of USC cell lines with both wild type and mutant p53. Taken together, our results suggest that metformin therapy could be a novel and attractive therapeutic approach for human USC, a highly aggressive variant of endometrial cancer.

## Introduction

Endometrial cancer is the most frequently occurring gynecologic cancer in Western countries. The incidence of the disease has been increasing in recent years, largely as a result of the growing obesity epidemic. However, treatment has remained relatively unchanged over the last 40 years, relying principally on surgery to achieve cure [1]. Endometrial cancers are classified into two major groups, with Type I being the most frequent (more than 80% of cases). Type I tumors are usually estrogen-dependent, low-grade neoplasms, with an endometroid, well-differentiated morphology, and are generally associated with a relatively good prognosis. On the other hand, Type II tumors are mostly diagnosed at an advanced stage, are not associated with exposure to estrogens, display a less differentiated phenotype, and have a worse prognosis. Uterine serous carcinoma (USC), which constitutes the predominant histological class among Type II tumors [2], is usually diagnosed at an advanced stage, and accounts for 50% of all relapses of the endometrial cancers, with a 5-year survival rate of 55%. The major genetic alterations that occur in Type I endometrial cancer include: microsatellite instability and mutations in the pTen, k-RAS and ß-catenin genes. On the other hand, Type II endometrial cancers have often p53 mutations, overexpression of Her2/neu oncogene and loss of heterozygosity on several chromosomes [3], [4]. Mutational analysis revealed that the USPC-2 cell line employed in the present study expresses a mutant p53 whereas USPC-1 cells express a wild type p53 (containing a number of polymorphisms) [2]. p53 is a tumor suppressor protein that regulates the expression of a wide variety of genes involved in apoptosis, growth arrest, inhibition of cell cycle progression, differentiation and accelerated DNA repair or senescence in response to genotoxic or cellular stress.

A number of studies have shown that patients with type 2 diabetes have an increased risk for certain types of cancer [5], including endometrial tumors [6]. Known risk factors for this disease include, in addition, obesity, hypertension, late menopause, and estrogen use [7]. Insulin resistant women generally carry excess body weight and are physically less active. In agreement with this notion, epidemiological evidence has shown that at least 40% of endometrial cancers can be attributed to excess body weight [8]. Evidence of an increased risk of cancer with diabetes and obesity has led to great concern given the worldwide epidemic of obesity and diabetes.

Metformin, (N, N-dimethylbiguanide), a safe oral anti-hyperglycemic agent of the biguanides family, is undergoing a renaissance because of its potential as a cancer therapy along with its traditional role in treating diabetes. Recent studies reported that metformin use was associated with a significant decrease in the incidence of cancer [9]. *In vitro* studies suggested that metformin inhibits cancer cell growth by activating adenosine monophosphate protein kinase (Ampk), by inactivating the mammalian target of rapamycin (mTOR), and also by decreasing the activity of the mTOR effector S6K1 [10], [11]. Furthermore, it has been demonstrated that inhibition of the mTOR pathway by rapamycin and its derivates leads to decreased protein synthesis and decreased cell proliferation in a number of experimental systems [12]–[15]. Rapamycin effectively inhibits the growth of ovarian tumors that rely on AKT signaling for proliferation, while tumors with alternative survival pathways may require the inactivation of multiple individual pathways for successful treatment [16]. Inhibition of ovarian cancer cells growth following treatment with metformin was reported recently [17], [18] and metformin was shown to potentiate the effect of cisplatin in these cells. Other studies revealed that metformin also induced a significant inhibition in proliferation, growth arrest and induction of apoptosis, and enhanced the sensitivity to chemotherapy in Type I endometrial cancer [19], [20].

The potential link between the insulin/IGF-I signaling pathways and cancer has been the focus of much investigation over the last several years [17], [21]–[23]. The biological actions of IGF-I are mediated by the IGF-I receptor (IGF-IR), a tyrosine kinase-containing heterotetramer with potent antiapoptotic activity. Regulation of IGF-IR gene expression is mainly mediated at the level of transcription and we have previously provided evidence that the IGF-IR gene constitutes a target for p53 action. Specifically, wild type p53 was shown to suppress IGF-IR promoter activity as well as endogenous IGF-IR mRNA levels whereas, in contrast, mutant forms of p53 enhanced IGF-IR gene expression [24].

Several studies have shown a correlation between components of IGF system and endometrial cancer risk. The IGF system plays an important role in the biology of endometrial cancer [17], [25]. Increased risk is related to higher levels of insulin and IGF-I. *In vivo* studies showed that increased insulin, IGF-I, and IGF-II signaling through the insulin receptor and IGF-IR can in fact induce tumorigenesis by up-regulating the insulin receptor and IGF-IR signaling pathways. A correlation between hyperinsulinemia, insulin resistance, and ovarian cancer development was demonstrated by Augustin et al [26]. *In vitro* and *in vivo* studies showed increased peripheral insulin sensitivity and cancer growth inhibition by Ampk activation [27]. Furthermore, a recent study reported that treatment with an mTOR inhibitor (WAY-129327) decreased endometrial proliferation, whereas mTOR activation was followed by loss of negative feedback to insulin receptor substrate-1 (IRS-1) during the early stages of cancer development [17]. In view of the interplay between the insulin/IGF-I and metformin signaling pathways, the aim of the current study was to assess the effect of metformin on USC and to evaluate the hypothesis that the mechanism of action of metformin may involve inhibition of the IGF-I pathway.

## Materials and Methods

### Cell Lines and Treatments

Human endometroid endometrial carcinoma cells (ECC-1 and Ishikawa; Type I) were obtained from Dr. Y. Sharoni (Ben Gurion University, Beer Sheba, Israel). Serous papillary (USPC-1 and USPC-2; Type II) endometrial cancer cell lines were kindly provided by Dr. A. Santin (Yale University School of Medicine, New Haven, CT, USA). USPC cells were grown in RPMI-1640 medium (Biological Industries Ltd., Beit-Haemek, Israel) [28]. Metformin was obtained from Sigma-Aldrich Ltd (St. Louis, MO, USA). In all of the experiments, cells were serum-starved for 24 h, after which they were treated with 10 mM metformin, in the presence or absence of IGF-I (50 ng/ml) (PeproTech Ltd, Rocky Hill, NJ, USA).

### Western Immunoblots

Cells were serum starved overnight and then incubated with metformin, in the presence or absence of IGF-I. After incubation, cells were harvested and lysed in a buffer containing protease inhibitors (Cell Signaling Technology, Beverly, MA, USA). Protein content was determined using the Bradford reagent (Bio-Rad, Hercules, CA, USA) and bovine serum albumin (BSA) as a standard. Samples were electrophoresed through 15%, 10% or 5% SDS-PAGE gels, followed by blotting of the proteins onto nitrocellulose membranes. After blocking with either 5% skim milk and/or 3% BSA, the blots were incubated overnight with the antibodies listed below, washed and incubated with the appropriate horseradish peroxidase (HRP)-conjugated secondary antibody. Antibodies against phospho-IGF-IR (3024), IGF-IR ß-subunit (3027), insulin receptor [(IR); 3025], phospho-AKT (9271), AKT (9272), phospho-ERK1/2 (9106), phospho-p53 (9284), poly ADP ribose polymerase [(PARP); 9542], caspase 3 (9661), pTen (9559), p21 (2947), phospho-GSK3ß (Ser9), Foxo1 (9462), phospho-Ampk (2531), Ampk (2532), phospho-mTOR (5536), mTOR (2983) and PI3 kinase p85 (4292) were obtained from Cell Signaling Technology. Antibodies against ERK1 (K-23), Sp1 (PEP2), E2F1 (KH95), retinoblastoma [Rb (C-15)], p53 (mixture of DO-1 and Pab 1801), cyclin D1 (H295) and caspase 9 (H-83) were purchased from Santa Cruz Biotechnology (Santa Cruz, CA, USA). An antibody against actin (Clone C4) was purchased from ICN Biomedicals, Inc. (Aurora, OH, USA) and anti-PAN-Ras (Ab-3) was from Oncogene Research Products (San Diego, CA, USA). In addition, a GSK3ß antibody (610201) from BD Transduction Laboratories (Franklin Lakes, NJ, USA) was used. The secondary antibodies were HRP-conjugated goat anti-rabbit IgG (1∶50,000) and donkey anti-mouse IgG (1∶25,000; Jackson ImmunoResearch Laboratories, West Grove, PA, USA). Proteins were detected using the SuperSignal West PicoChemiluminescent Substrate (Pierce, Rockford, IL, USA). The expression of actin was used as a loading control of total proteins.

### Transfections and Luciferase Assays

IGF-IR and insulin receptor (IR) promoter luciferase reporter constructs were employed for transient transfection experiments. The IGF-IR promoter construct includes 476 bp of the 5′-flanking and 640 bp of the 5′-untranslated regions of the IGF-IR gene (p[−476/+640] luciferase [LUC]) [28], [29]. The IR promoter construct (gift from Dr. Antonio Brunetti, Catanzaro, Italy) includes the region from −2 bp to −1823 bp upstream of the translation initiation site [30]. USPC-1 and USPC-2 cells were transfected as previously described [28], [29]. Metformin was added to the medium during the last 24 h of the incubation. Cells were harvested 48 h after transfection and luciferase and β-galactosidase activities were measured as previously described [28]. Promoter activities were expressed as luciferase values normalized for ⇓-galactosidase activity.

### Proliferation Assays

Cells were seeded onto 24-well plates (5×10^4^ USPC-1 cells/well and 3.6×10^4^ USPC-2 cells/well). After 24 h, the cells were treated with metformin for 24, 48, or 72 h, respectively, in triplicate wells. Cell viability was assessed using a standard Thiazolyl Blue Tetrazolium Bromide (MTT) method [28]. Cell viability was expressed as a percentage of optical density values obtained upon treatment relative to controls.

### Cell Cycle Analysis

Cells were seeded onto 6-well plates (1×10^6^ cells/well) for 24 h. Cells were then serum-starved for an additional 24 h and incubated in the presence or absence of metformin for 72 h. After incubation, cells were washed with phosphate-buffered saline, trypsinized, centrifuged, resuspended in citrate buffer and stored at −80°C prior to analysis. The cells were thawed and permeabilized before adding propidium iodide according to Vindelov et al [31]. Stained cells were analyzed using a FacsCalibur system (Cytek Development Inc, Fremont, CA, USA).

### Wound-healing Assays

Wound-healing assays were performed as described previously [28]. Briefly, USPC-1 and USPC-2 monolayers were grown to confluence in 6-well plates, after which a wound was made in the cell monolayer using a sterile micropipette tip. Then, cells were cultured in starvation medium, in the presence or absence of IGF-I, in combination with metformin. At 0 h the scratched monolayer cultures were photographed using an inverted microscope (ECLIPSE Ti, Nikon Corporation, USA). Cell movement was assessed 48, 72, and 96 h after wounding from photographs taken under the microscope with a 10× objective. Distance of cell migration was measured at middle position on the screen using Microsoft Windows PowerPoint.

## Results

### Effect of Metformin on the IGF-I Signaling Pathway

**Figure 1:**
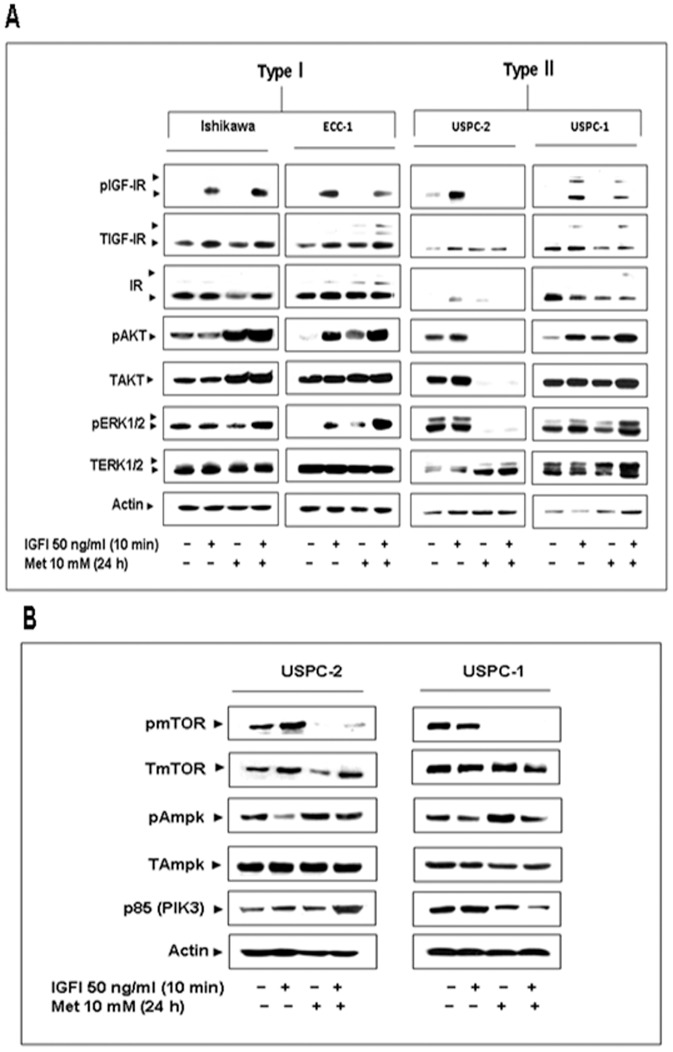
Effect of metformin on IGF-I-mediated signal transduction and mTOR and Ampk signalling pathway in endometrial cancer cells. A, Ishikawa, ECC-1, USPC-2 and USPC-1 cells were treated with metformin (10 mM) for 24 h (or left untreated) in the presence or absence of IGF-I (50 ng/ml) during the last 10 min of the incubation period. Whole cell lysates (100 µg) were resolved by SDS-PAGE and immunoblotted with antibodies against pIGF-IR, TIGF-IR, IR, pAKT, TAKT, pERK1/2, TERK1/2 and actin, followed by incubation with an HRP-conjugated secondary antibody. The figure shows the results of a typical experiment, repeated three times with similar results. B, USPC-2 and USPC-1 cell lines were treated with metformin for 24 h (or left untreated) and/or IGF-I during the last 10 min of the incubation. Whole cell lysates (100 µg) were resolved by SDS-PAGE and immunoblotted with antibodies against pmTOR, TmTOR, pAmpk, TAmpk, and p85. The figure shows the results of a typical experiment, repeated three times with similar results.

To examine the potential regulation of the expression and activation of IGF-IR and downstream signaling mediators by metformin in different types of endometrial carcinoma, Type I (Ishikawa, ECC-1) and Type II (USPC-2, USPC-1) cancer cells were treated with metformin for 24 h, in the presence or absence of IGF-I during the last 10 min (Figure 1A). Results of Western blots showed that metformin decreased the IGF-I-stimulated phosphorylation of IGF-IR in ECC-1, USPC-2 and USPC-1 cells, whereas it slightly up-regulated IGF-IR phosphorylation in Ishikawa cells. Furthermore, metformin up-regulated AKT and ERK1/2 phosphorylation in Ishikawa, ECC-1 and USPC-1 cells, while it down-regulated AKT and ERK1/2 phosphorylation in USPC-2 cells. In addition, metformin down-regulated the expression of total IGF-IR and IR in USPC-2 and USPC-1 cells. Metformin did not alter ERK1/2 expression in neither cell line. A summary of scanning densitometry results is presented in Table 1.

**Table 1:** Scanning densitometry analysis of the effect of metformin on IGF-I-stimulated IGF-IR, AKT and ERK1/2 phosphorylation.

	Ishikiwa				ECC-1				USPC-1				USPC-2			
	–	I	M	M+I	–	I	M	M+I	–	I	M	M+I	–	I	M	M+I
pIGF-IR/TIGF-IR	0	100	0	132	0	100	0	41	47	100	0	0	0	100	0	85
pAKT/TART	118	100	150	156	58	100	63	121	100	100	0	0	25	100	84	124
pERK1/2/TERK1/2	121	100	75	180	0	100	56	322	152	100	18	0.9	56	100	51	82

Optical density was expressed as pIGF-IR, pAKT and pERK values normalized to the corresponding total proteins. A value of 100% was given to the optical density of IGF-I treated cells. The table shows the result of a typical experiment, repeated three times with similar results.

−, untreated cells; I, IGF-I-treated cells; M, metformin-treated cells; M+I, metformin and IGF-I-treated cells.

### Effect of Metformin on the mTOR and Ampk Pathway

Subsequent analyses focused on Type II endometrial cancer cells. Previous studies showed that metformin phosphorylates the inhibitory Ser^789^ site of IRS-1 *via* Ampk activation [32]. This was associated with decreased AKT activation [33], which led to reduced mTOR activation [23], [34]. To assess the effect of metformin on the mTOR and Ampk pathway in USC, cells were treated with metformin in the presence or absence of IGF-I, and mTOR, Ampk and PI3K levels were measured by Western blots. Metformin decreased phospho-mTOR levels in USPC-2 and USPC-1 cells, both in the presence or absence of IGF-I (Figure 1B). In addition, IGF-I decreased phospho-Ampk levels in both cell lines, whereas metformin increased the stimulated and unstimulated phosphorylation of Ampk. Moreover, metformin decreased p85 levels in USPC-1 cells, while it up-regulated p85 levels in USPC-2 cells only in combination with IGF-I. No consistent changes in total Ampk and mTOR levels were seen in neither cell line.

### Effect of Metformin on IGF-IR and IR Promoter Activities

**Figure 2:**
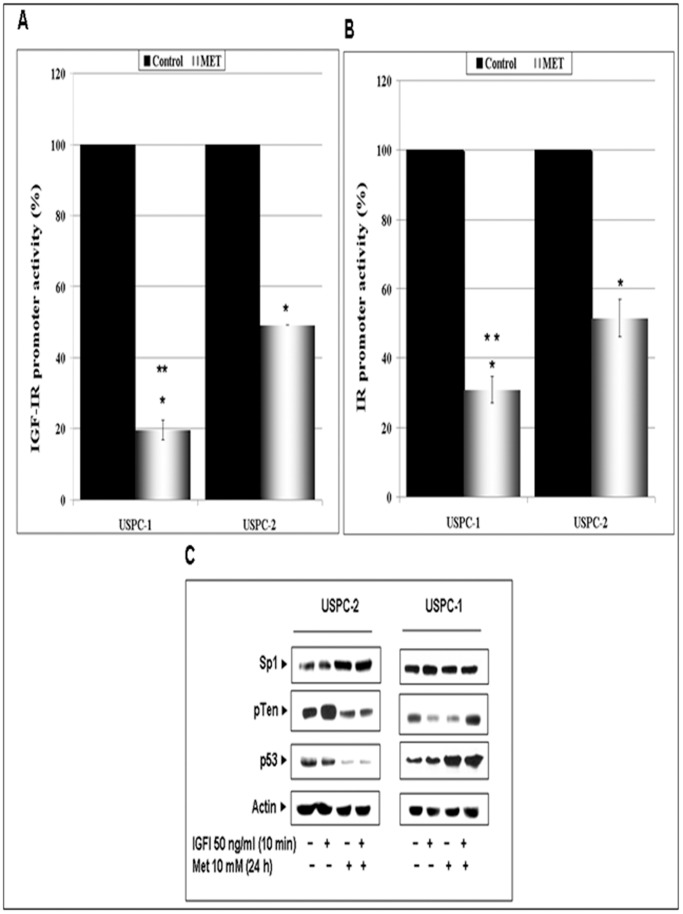
Regulation of IGF-IR and IR promoter activities and transcriptional activators by metformin in USC cells. USPC-1 and USPC-2 cells were transiently transfected with an IGF-IR promoter-luciferase reporter plasmid, p(-476/+640)LUC (A), or an IR promoter-luciferase reporter construct (B), along with a ß-galactosidase expression plasmid. Promoter activities were expressed as luciferase values normalized for ⇓-galactosidase activity. Results are mean ± SEM (duplicates samples of three independent experiments). *, p<0.05 versus untreated cells; **, p<0.05 versus USPC-2 cells transfected with IGF-IR or IR promoter luciferase constructs. C, Western blot analysis of Sp1, pTen, and p53 in USPC-2 and USPC-1 cells treated with metformin (24 h) and/or IGF-I (10 min). Whole-cell lysates (100 µg) were resolved by SDS-PAGE and immunoblotted with the indicated antibodies. Results are representative of three independent experiments.

To determine whether the effects of metformin on IGF-IR and IR gene expression were correlated with corresponding changes in promoter activities, USPC-1 and USPC-2 cells were transfected with luciferase reporter genes under the control of the IGF-IR or IR promoters. Twenty-four hours after transfection, metformin (10 mM or vehicle) was added to the medium and incubated for an additional 24 h. Forty-eight hours after transfection, the cells were collected and promoter activities were measured. The results obtained indicate that metformin repressed IGF-IR and IR promoter activities in both cell lines. Specifically, metformin decreased IGF-IR-promoter activity to 49±0.3% of control values in USPC-2 and to 20±2.8% in USPC-1 cells (Figure 2A). In addition, metformin suppressed IR promoter activity to 52±5.4% of control values in USPC-2 and to 31.3±2.2% in USPC-1 cells (Figure 2B). These data indicate that metformin reduced IGF-I and IR promoter activities in both cell lines.

### Effect of Metformin on Transcription Factors Involved in IGF-IR Gene Regulation

Given the inhibitory effect of metformin on IGF-IR promoter activity, we next measured the expression of a number of transcriptional activators shown to participate in IGF-IR gene modulation. Specifically, we investigated the effect of metformin on tumor suppressors p53 and pTEN and on zinc finger protein Sp1 expression. The rationale for these measurements was the fact that both p53 and pTEN down-regulate IGF-IR levels [35], [36], whereas Sp1 was identified as a potent IGF-IR transactivator [35]. For this purpose, cells were treated with metformin in the presence or absence of IGF-I, and p53, pTEN and Sp1 expressions were analyzed by Western blots. The data obtained revealed that metformin decreased pTEN and p53, and increased Sp1 levels in USPC-2 cells (Figure 2C). On the other hand, metformin up-regulated p53 levels in USPC-1 cells. A summary of scanning densitometry results is presented in Table 2.

**Table 2:** Scanning densitometry analysis of the effect of metformin on pTen, p53 and specific cell cycle regulatory proteins.

	USPC-1				USPC-2			
	−	I	M	M+I	−	I	M	M+I
pTen/Actin	63	100	48	43	236	100	114	339
p53/Actin	144	100	19	17	63	100	167	150
Cyclin D1/Actin	68	100	0	0	82	100	22	24
p21/Actin	85	100	0	0	148	100	73	85
Ras/Actin	87	100	206	312	219	100	9	32
Rb/Actin	118	100	220	184	108	100	100	174
E2F1/Actin	129	100	79	0	40	100	264	340

Optical density was expressed as pTen, p53, cyclin D1, p21, Ras, Rb and E2F1 values normalized to the corresponding actin levels. A value of 100% was given to the optical density of IGF-I treated cells. The table shows the result of a typical experiment, repeated three times with similar results.

−, untreated cells; I, IGF-I-treated cells; M, metformin-treated cells; M+I, metformin and IGF-I-treated cells.

### Effect of Metformin on the Induction of Apoptosis

**Figure 3:**
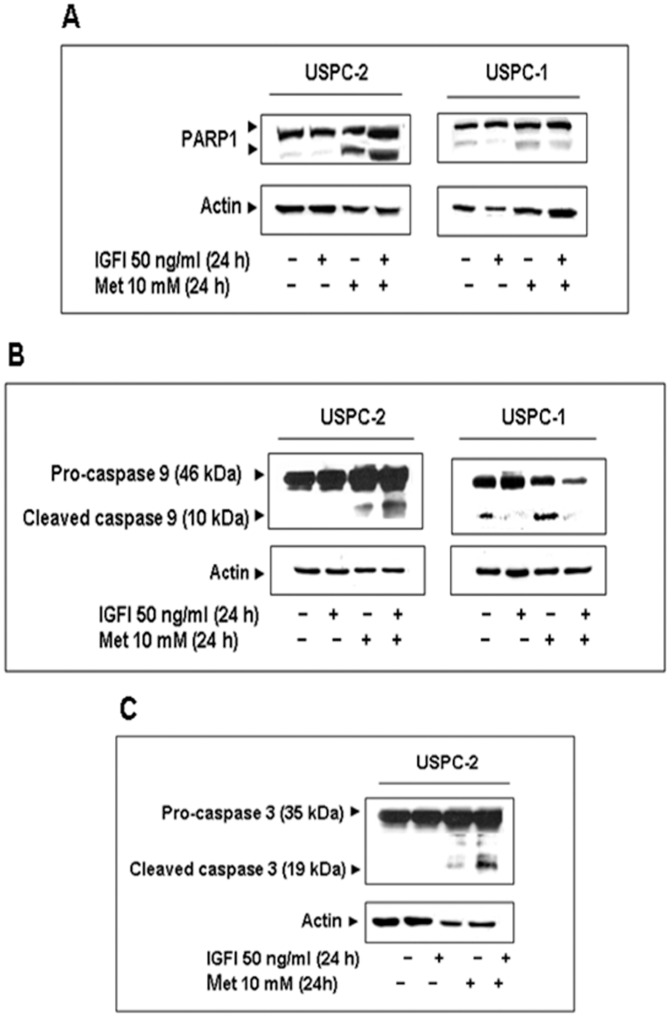
Effect of metformin on apoptosis. A, Western blot analysis of PARP1 in USPC-2 and USPC-1 cells. B, Western blot analysis of caspase 9 in USPC-2 and USPC-1 cells. C, Western blot analysis of caspase 3 in USPC-2 cells. Cells were treated with metformin for 24 h in the presence or absence of IGF-I. Whole-cell lysates (100 µg) were resolved by SDS-PAGE and immunoblotted with the indicated antibodies. Results are representative of three independent experiments.

After identification of some of the signaling pathways involved in metformin action in USC, we next explored the potential effect of metformin on apoptosis. To this end, USPC-2 and USPC-1 cells were serum-starved for 24 h and then treated with metformin, in the presence or absence of IGF-I, for 24 h. Apoptosis was detected by cleaved caspase-9 and PARP1 measurements using Western blots. Caspase-9 is an initiator caspase which, following activation, cleaves procaspases-3 and -7, with ensuing cleavage of several cellular targets, including PARP1. PARP1 serves as a substrate for both caspases-3 and -7 and cleaved PARP1 (∼85 kDa) is a hallmark of caspase-dependent apoptosis [28]. Western blots revealed that metformin treatment led to a large increase in cleaved PARP1 in USPC-2 cells and to a more modest, although still marked, increase in PARP1 cleavage in USPC-1 cells (Figure 3A). In addition, metformin induced a significant increase in cleaved caspase-9 in both cell lines. Addition of IGF-I along with metformin prevented the metformin-induced cleavage of pro-caspase-9 in USPC-1 cells (Figure 3B). To further investigate the molecular mechanisms that control apoptosis in USC, caspase-3 immunoblotting analyses were carried out in metformin-treated cells. Metformin-treated USPC-2 cells exhibited a marked increase in cleaved caspase-3 levels (Figure 3C), although no effect was seen in USPC-1 cells (data not shown).

### Effect of Metformin on Cell Proliferation/survival

**Figure 4:**
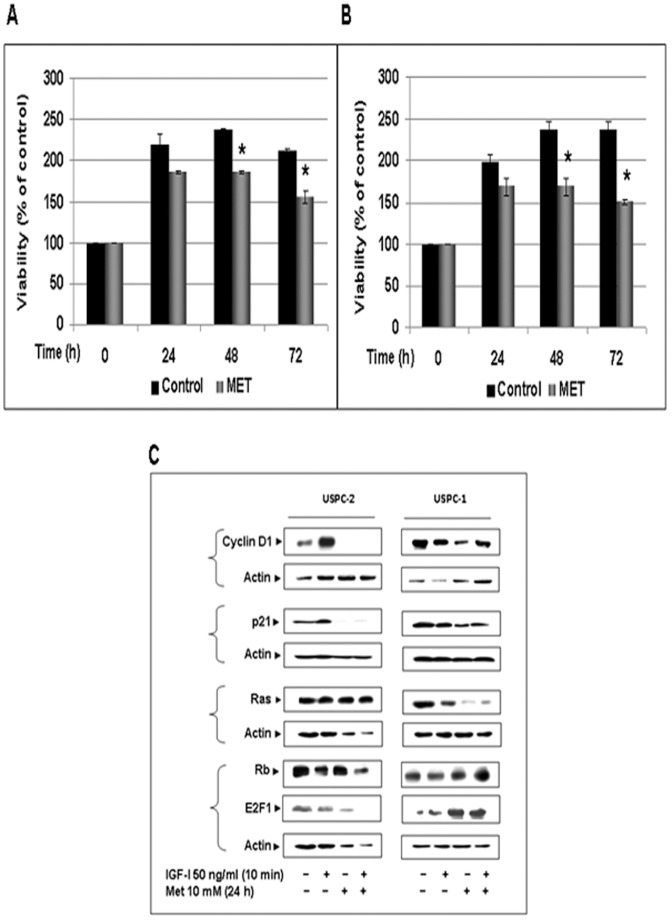
Effect of metformin on proliferation and cell cycle regulatory proteins in USC cells. Cells were plated in 24-well plates at a density of 5×10^4^ cells/well for USPC-2 (A) and 3.6×10^4^ cells/well for USPC-1 (B). Cells were incubated in the absence (open bars) or presence (solid bars) of metformin, and proliferation was evaluated at 24, 48 and 72 h by MTT measurements. A value of 100% was given to the cell number at time 0. The bars represent the mean ± S.E.M. of three independent experiments, performed each in triplicate samples; *p<0.05 versus untreated cells. C, Western blot analysis of cyclin D1, p21, Ras, Rb and E2F1 in USPC-2 and USPC-1 cells treated with metformin for 24 h in the absence or presence of IGF-I. Whole-cell lysates (100 µg) were resolved by SDS-PAGE and immunoblotted with the indicated antibodies. Results are representative of three independent experiments.

To evaluate the potential anti-proliferative effect of metformin, USPC-2 and USPC-1 cells were grown in 10% FBS-containing media with metformin for 24, 48, and 72 h. The proliferation rates were determined by MTT assays. Results obtained showed that addition of metformin led to a significant decrease in proliferation compared with untreated (control) cells. Thus, in USPC-2 cells proliferation rates in the presence of metformin were 85±1.6%, 78±2%, and 73.5±8.3% of controls at 24, 48, and 72 h, respectively (Figure 4A). In USPC-1 cells, proliferation rates in the presence of metformin were 85±10%, 71±10%, and 63.4±2.9% of controls at the same time points (Figure 4B).

### Effects of Metformin on Cell Cycle Progression

Given the metformin-mediated inhibition of cell proliferation, experiments were carried out next to characterize the effect of metformin on cell cycle progression. To this end, USPC-2 and USPC-1 cells were treated with metformin for 72 h, after which flow cytometry was performed on propidium iodide-stained cells. In USPC-1 cells, metformin treatment increased the proportion of cells at the G0/G1 phase from 87.22±0.7% to 91±0.49% and decreased the number of cells at S phase from 8±0.164% to 5.2±0.09% and at the G2/M phase from 4.78±0.59% to 3.8±0.51%. In contrast, in USPC-2 cells metformin led to a decrease in the proportion of cells at the G0/G1 phase from 86±0.6% to 73±1.2% and an increase in the portion of cells at S phase from 5±0.6% to 12±0.12% and in G2/M phase from 9±0% to 15±0.3%. The data obtained indicate that metformin inhibited cell cycle progression in USPC-1 cells (containing a wild type p53). However, it had a paradoxical effect in USPC-2 cells (containing a mutant p53), as indicated by increases in the proportion of cells at the S and G2/M phases (Table 3).

**Table 3:** Effect of metformin on the cell cycle in USC.

Cells	Control	Metformin
USPC-1		
G_0_/G1	87.22±0.7	91±0.49* (4%↑)
S	8±0.164	5.2±0.09* (35%↓)
G_2_	4.78±0.59	3.8±0.51 (20%↓)
**USPC-2**		
	**Control**	**Metformin**
G_0_/G1	86±0.6	73±1.2* (15.1%↓)
S	5±0. 6	12±0.12* (60%↑)
G_2_	9±0	15±0.3* (67%↑)

USPC-2 and USPC-1 cells were seeded in quadruplicate dishes, serum-starved for 24 h, and treated with metformin (or left untreated, controls) for 72 h. Cell cycle distribution was assessed by FACS analysis. The values in the table denote mean ± SEM;

* p<0.05 versus control cells.

### Effects of Metformin on Cell Cycle Regulatory Proteins

Next, Western blotting was used to examine the effects of metformin on various cell cycle regulatory proteins, including cyclin D1, cyclin-dependent kinase (CDK) inhibitor p21, Ras, Rb, and E2F1. Results obtained showed that metformin, both in the presence or absence of IGF-I, markedly down-regulated cyclin D1 and p21 levels in both cell lines, and down-regulated Ras expression only in USPC-1 cells (Figure 4C). Next, the metformin effect on downstream targets of CDKs, including transcription factor E2F1 and its regulator, Rb, were examined. Data obtained revealed that E2F1 levels were decreased in USPC-2 and increased in USPC-1 cells following metformin treatment, both in the presence or absence of IGF-I. In addition, metformin treatment resulted in the accumulation of p53 protein in wild type p53-containing USPC-1, but not in mutant p53-containing USPC-2, cells (Figure 2C). A summary of scanning densitometry results is presented in Table 2.

### Effect of Metformin on Cell Migration

**Figure 5:**
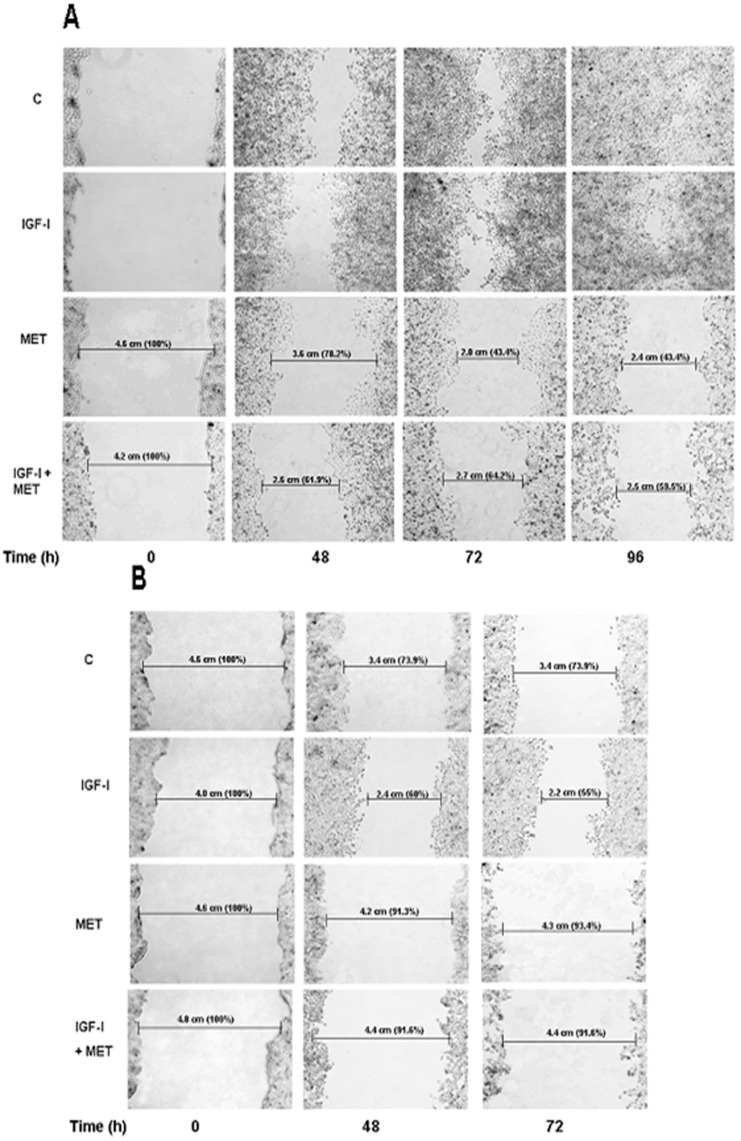
Effect of metformin on cell migration. Wounds were made on monolayers of USPC-2 (A) and USPC-1 (B) cells grown to 100% confluence. Cells were then incubated in serum-free media containing IGF-I (50 ng/ml), metformin (10 mM), or both, for 48, 72 and 96 h (USPC-2) and for 48 and 72 h (USPC-1). Treated or untreated (control) cells were photographed just after scratch (time 0), and after 48, 72 and 96 h. Results presented here are representative of triplicate independent samples of each cell line. The rate of migration was measured by quantifying the total distance that the cells (as indicated by rulers) moved from the edge of the scratch toward the centre of the scratch. A value of 100% was given to the wound area at time 0. The migration of IGF-I and/or metformin treated samples was compared to wound area at time 0.

Wound-healing assays were performed to investigate the potential inhibitory effect of metformin on cell migration in USC. For this purpose, USPC-2 and USPC-1 cells were incubated in serum-free media containing IGF-I, metformin, or both, for 48, 72, and 96 h (USPC-2) or 48 and 72 h (USPC-1). As illustrated in Figures 5A and B, results of wound-healing assays indicate that the migration of both cells were inhibited by metformin, both in the presence and absence of IGF-I. In addition, data obtained showed that both untreated and IGF-I-treated USPC-2 and USPC-1 cells migrated at similar rates over 96 h (USPC-2) and 72 h (USPC-1).

### Effect of Metformin on Glycogen Synthase Kinase-3⇓ (GSK3⇓) Expression

**Figure 6:**
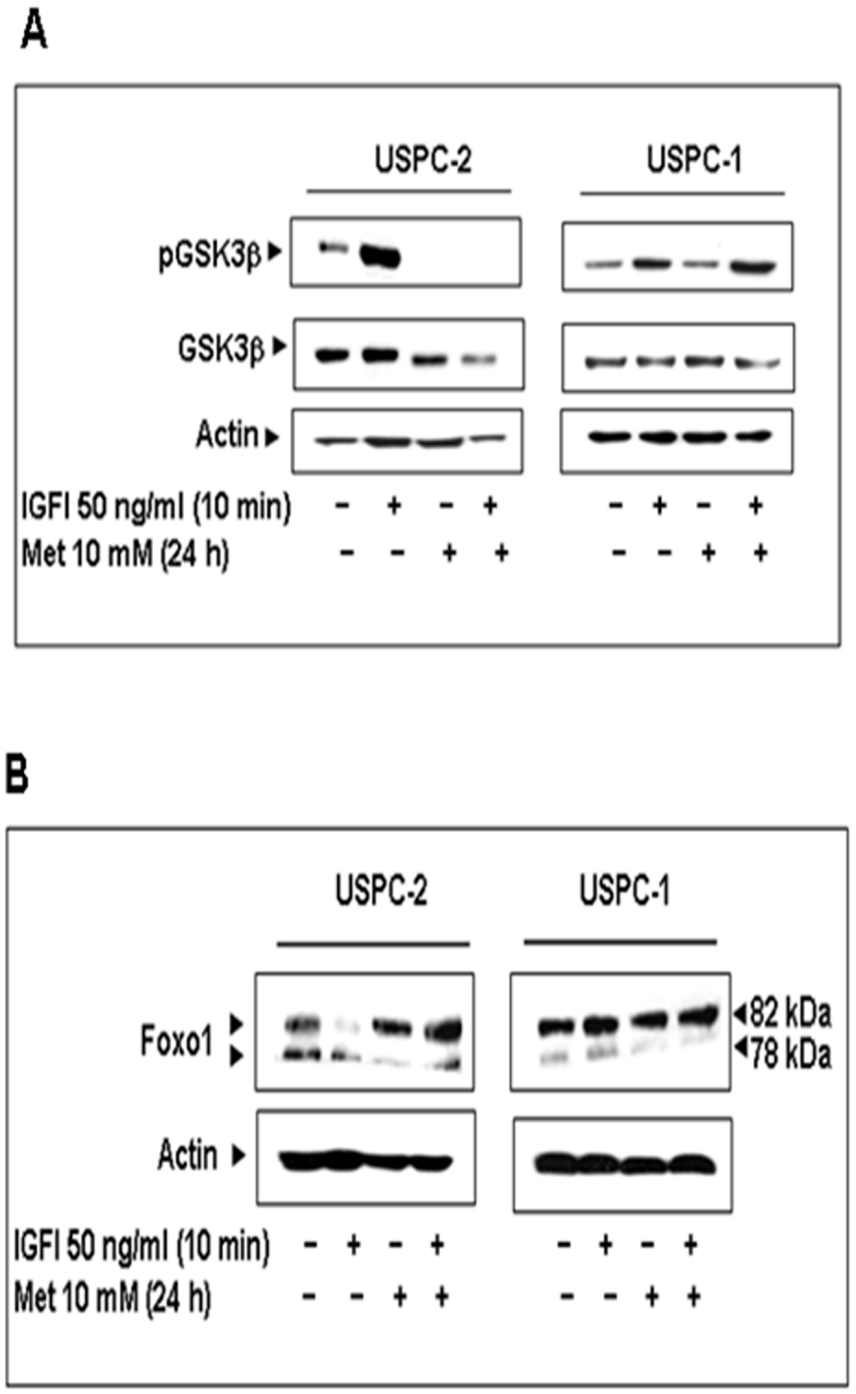
Effect of metformin on GSK3ß and Foxo1 expression. A, Western blot of pGSK3ß and GSK3ß in USPC-2 and USPC-1 cells treated with metformin for 24 h and/or IGF-I. The figure shows the results of a typical experiment repeated three times. B, Western blot analysis of Foxo1 on USPC-2 and USPC-1 cells treated for 24 h with metformin and/or IGF-I. The figure shows the results of a characteristic experiment, repeated three times with similar results.

Glycogen synthase kinase-3ß (GSK3ß) is a Ser/Thr kinase that has been identified as a regulator of glycogen metabolism [37]. In addition, studies have shown that GSK3 is implicated in both insulin action and adipogenesis [38]. To address the metabolic effect of metformin in USC, we examined the effect of metformin on GSK3ß expression and activation in USPC-2 and UCPC-1 cells. Results of Western blots showed that metformin decreased the basal and IGF-I stimulated phosphorylation of GSK3ß and downregulated GSK3ß levels in USPC-2 cells (Figure 6A). No changes in GSK3ß expression or phosphorylation were seen in USPC-1 cells.

### Effect of Metformin on Forkhead Transcription Factor (Foxo1)

Finally, recent studies have shown that metformin reduces lipid accumulation in macrophages by inhibiting Foxo1-mediated transcription of fatty acid-binding protein 4 [39]. To establish the effect of metformin on Foxo1 levels as a marker of lipogenesis, USPC-2 and USPC-1 cells were treated with metformin in the presence and absence of IGF-I. Western blots revealed that metformin decreased Foxo1 levels in both cell lines (Figure 6B).

## Discussion

USC is an aggressive subtype of endometrial cancer. Although less common than its endometrioid carcinoma counterpart, USC accounts for a disproportionate number of endometrial cancer-related recurrences and subsequent deaths. Therefore, developing targeted approaches to treat this aggressive form of endometrial cancer has a high priority. Metformin is an anti-diabetic drug with potential anti-neoplastic actions. To date, no studies have addressed the activity of metformin in USC. Studies have shown a correlation between obesity and endometrial cancer risk. For example, Libby et al [40], in an observational cohort study, found that metformin use was associated with 37% lower adjusted incidence of cancer. Currie et al [41], in a retrospective cohort study, found that metformin treatment was associated with lower risk of cancer, compared to other glucose-lowering therapies. In addition, improved response to chemotherapy was seen in diabetic breast cancer patients receiving metformin, as opposed to those not receiving the drug [42].

Recent studies demonstrated a significant antiproliferative activity of metformin in Type I endometrial cancer. Cantrell et al [19] reported that metformin treatment resulted in G1 arrest, induction of apoptosis and decrease in cell proliferation in ECC and Ishikawa endometrial cells. This effect was partially mediated through Ampk activation and subsequent inhibition of the mTOR pathway. Furthermore, recent studies showed that metformin enhanced the sensitivity of type I endometrial cells to cisplatin and taxol chemotherapy [43], [44]. The mechanisms of action of IGF-I are interconnected to the insulin signaling pathways. In this study we demonstrated that metformin effectively blocked IGF-IR activity in three of the endometrial cancer cells lines assayed (ECC-1, USPC-2 and USPC-1), while a slight increase in IGF-IR activation was seen in Ishikawa cells. In addition, metformin down-regulated the expression of IGF-IR and IR in USPC-1 and USPC-2 cells, and up-regulated IGF-IR and IR levels in Ishikawa and ECC-1 cells. Furthermore, metformin did not decrease ERK and AKT phosphorylation in Ishikawa and ECC-1 cells. Part of these, seemingly, paradoxical results can be explained by the fact that Ishikawa cells are known to have mutations in the *ras* proto-oncogene, p53 [45] and pTen tumor suppressor gene, leading to constitutive phosphorylation of AKT [46]. Interestingly, Ishikawa cells secrete IGF-II, but not IGF-I [47]. pTen is known to downregulate IGF-II and IGF-IR expression in hepatoma and prostate cancer cells [48], [49], suggesting that the expression of a constitutively-active AKT in Ishikawa cells may induce an increase in IGF-IR expression. Unlike Ishikawa, ECC-1 cells are not well characterized but exhibit the same p53 mutation as Ishikawa cells [50]. As mentioned above, it is possible that the increased levels of IGF-IR in ECC-1 ‘prompted’ the cells to develop alternative mechanisms to activate the MAPK and PI3K pathways that could not be blocked at the level of the IGF-IR.

USPC-1 and USPC-2 cells are regarded as a validated model for USC [51]. The USPC-1 cell line was generated from a grade IV biopsy from a 65-year-old white patient, whereas the USPC-2 line was derived from a grade III biopsy from a 75-year-old African-American patient. USPC-1 cells express a wild type p53 including two polymorphisms (deletion of 16 bp in intron 3 [c.96+41del16bp] and a C>A transition in exon 4, position 29). USPC-2 cells, on the other hand, exhibit a mutation in exon 5 (position c.493), which results in the formation of a stop codon at position p.165. In addition, USPC-1 cells express higher endogenous IGF-IR and IR protein and mRNA levels than USPC-2 cells [52]. We have recently shown that wild type, but not mutant, p53 represses IGF-IR promoter activity in USC cell lines, suggesting that IGF-IR levels are strongly dependent on p53 status [2]. Our present data demonstrate that metformin increases wild type p53 levels in USPC-1 cells, whereas it decreases mutant p53 levels in USPC-2 cells. These results suggest that mutant p53 is, most probably, inactivated and, therefore, unable to repress IGF-IR and IR promoters. The inhibition of IGF-IR/IR promoter activities seen in both cells is most probably explained by the fact that the transcription regulatory machineries involve a number of nuclear proteins, including WT1, KLF6, and/or multiprotein complexes [35], and it is often difficult to dissect the contribution of individual transcription factors.

In addition, our results revealed that metformin caused a progressive accumulation of USPC-1, but not USPC-2, cells in G0/G1, with a marked decrease in the percentage of cells in S and G2/M phases. The cyclin D1/CDK complex is responsible for the binding and sequestration of p27Kip1 and p21Cip1. This prevents these proteins from binding to, and inhibiting the cyclin E/CDK2 complex, which promotes progression from G0/G1 to S phase of the cell cycle [53]. Given that metformin causes downregulation of cyclin D1, this may lead to release of p27Kip1 and p21Cip1, allowing them to bind to CDK2, hence blocking its activity. Taken together, our data suggest that inhibition of cell proliferation and growth arrest at the G1-S stage by metformin is dependent on cyclin D1 downregulation followed by enhanced binding of the cyclin E/CDK2 complex by p21 and/or p27 in USPC-1 cells. In the presence of a wild type p53 (USPC-1 cells), this accumulation causes growth arrest and apoptosis, but in the presence of a nonfunctional p53 (USPC-2 cells), no growth arrest occurs. The metformin-induced apoptosis in USPC-2 cells was associated with activation of caspases-3 and -9. The activation of caspase-3 was accompanied by PARP cleavage into an 85-kDa fragment. We propose that the metformin-induced apoptosis in USPC-2 cells is mediated *via* a p53-independent mechanism, whereas the metformin-induced apoptosis in USPC-1 constitutes, most probably, a p53-dependent event. Metformin-induced apoptosis in USPC-1 cells might be related to caspase-9 activation, accompanied by PARP cleavage. The role of p53 in metformin action is controversial and appears to be cell type-specific. Previous investigations showed that p53-deficient colon cancer cells, but not wild-type p53-containing cells, are sensitive to metformin. On the other hand, studies demonstrated that cancer cells with a functional p53 (e.g., LNCaP, MCF-7) exhibited a similar sensitivity to metformin than p53-null cells [54]. In colon cancer cells, metformin was found to induce apoptosis *in vitro* only in cells lacking p53 [19]. Hence, lack of p53 may facilitate metformin’s pro-apoptotic action whereas, on the other hand, p53 may be required to mediate metformin’s action on cell cycle arrest. We found that metformin can mediate apoptosis in USPC-2 cells *via* a p53-independent mechanism, but is unable to elicit cell cycle arrest. In contrast, metformin can mediate both effects through a p53-dependent mechanism in USPC-1 cells. More studies are required to elucidate the role of p53 in metformin action.

Results of MTT assays are consistent with an anti-proliferative activity of metformin. We speculate that the metformin-induced activation of Ampk and inhibition of cell growth might be mediated *via* the PI3K/AKT pathway in a p53-independent manner in USPC-2 cells and by p53/Ampk signalling pathways in USPC-1 cells. Cantrell et al [19] recently showed that metformin inhibits Type I endometrial cancer cell proliferation and that this effect was partially mediated by inhibition of the mTOR pathway. Xie et al [20] showed that metformin promotes progesterone receptor (PR) expression in endometrial cancer, whereas IGF-I and IGF-II inhibit PR expression. This effect was partially mediated through inhibition of the mTOR pathway. Normal cell growth depends on a balance between the Ampk/AKT and the Rapamycin (mTOR) pathways [19], [55]. Deregulation of this fine balance might lead to metabolic growth related diseases, resistance to apoptosis, and increased proliferation [55], [56]. Under normal circumstances, upstream receptor tyrosine kinases, especially the IGF-I pathway, regulate this pathway [57]. Interestingly, it has been recently suggested that inhibition of mTOR could induce the release of feedback inhibition, paradoxically activating IGF-I signalling, and thus reducing the effectiveness of the mTOR inhibitors [23]. Results of migration assays suggest that both cell lines secrete a variety of growth factors and cytokines that may act as paracrine and/or autocrine regulators of proliferation [8], [51].

The effect of metformin on glycogen synthesis in endometrial cancer has not yet been explored. GSK-3ß is a ubiquitous kinase implicated in both insulin action and adipogenesis [38]. GSK3ß was the first identified substrate of AKT and is usually constitutively active. Activated AKT phosphorylates GSK3ß Ser^9^, leading to its inactivation. As an upstream apoptotic regulator, GSK3ß is involved in regulation of apoptosis in different cells [58], [59]. In addition to its role in glucose metabolism, GSK3ß is also a key regulator of multiple processes, such as embryo development, cytoskeletal organization, protein synthesis, and adipogenesis [38]. Our data showed that metformin decreases the IGF-I-stimulated phosphorylation of AKT and GSK3ß in USPC-2 cells, while increasing their phosphorylation in USPC-1 cells. In addition metformin downregulated GSK3ß levels in USPC-2 cells. Potential associations between changes in total GSK3ß levels and IR and/or IGF-IR levels need to be further investigated. Finally, metformin has been reported to reduce lipid accumulation in adipocytes. In the present study, we established that metformin represses Foxo1 expression both in the presence or absence of IGF-I [39].

In summary, our study demonstrates that metformin displays potent apoptotic and anti-mitogenic actions in USC cells that are mediated, at least in part, *via* interaction with the IGF-IR axis. The inhibitory activities of metformin were observed in cells containing a wild type p53 gene as well as in cells expressing a mutant p53, suggesting that metformin actions are, most probably, not dependent on p53 status. Taken together, our results suggest that metformin might constitute a promising therapeutic agent for uterine serous carcinoma, with p53 genotype probably affecting outcome in a subset of tumors.

## References

[1] Kitchener H (2006) Management of endometrial cancer. Eur J Surg Oncol 32: 838–843. 16765558 10.1016/j.ejso.2006.03.046

[2] Attias-Geva Z, Bentov I, Kidron D, Amichay K, Sarfstein R, et al (2012) p53 Regulates insulin-like growth factor-I receptor gene expression in uterine serous carcinoma and predicts responsiveness to an insulin-like growth factor-I receptor-directed targeted therapy. Eur J Cancer 48: 1570–1580. 22033326 10.1016/j.ejca.2011.09.014

[3] Pallares J, Martinez-Guitarte JL, Dolcet X, Llobet D, Rue M, et al (2004) Abnormalities in the NF-kappa B family and related proteins in endometrial carcinoma. J Pathol 204: 569–577. 15481028 10.1002/path.1666

[4] Ryan AJ, Susil B, Jobling TW, Oehler MK (2005) Endometrial cancer. Cell Tissue Res 322: 53–61. 15947972 10.1007/s00441-005-1109-5

[5] Landman GW, van Hateren KJ, Kleefstra N, Groenier KH, Gans RO, et al (2010) Health-related quality of life and mortality in a general and elderly population of patients with type 2 diabetes (ZODIAC-18). Diabetes Care 33: 2378–2382. 20805257 10.2337/dc10-0979 PMC2963498

[6] Soliman PT, Wu D, Tortolero-Luna G, Schmeler KM, Slomovitz BM, et al (2006) Association between adiponectin, insulin resistance, and endometrial cancer. Cancer 106: 2376–2381. 16639730 10.1002/cncr.21866

[7] Liu FS (2007) Molecular carcinogenesis of endometrial cancer. Taiwan J Obstet Gynecol 46: 26–32. 17389185 10.1016/S1028-4559(08)60102-3

[8] Kaaks R, Lukanova A, Kurzer MS (2002) Obesity, endogenous hormones, and endometrial cancer risk: A synthetic review. Cancer Epidemiol Biomarkers Prev 11: 1531–1543. 12496040

[9] Rocha GZ, Dias MM, Ropelle ER, Osorio-Costa F, Rossato FA, et al (2011) Metformin amplifies chemotherapy-induced AMPK activation and antitumoral growth. Clin Cancer Res 17: 3993–4005. 21543517 10.1158/1078-0432.CCR-10-2243

[10] Hadad SM, Fleming S, Thompson AM (2008) Targeting AMPK: a new therapeutic opportunity in breast cancer. Crit Rev Oncol/Hematol 67: 1–7. 10.1016/j.critrevonc.2008.01.007 18343152

[11] Alimova IN, Liu B, Fan Z, Edgerton SM, Dillon T, et al (2009) Metformin inhibits breast cancer cell growth, colony formation and induces cell cycle arrest in vitro. Cell Cycle 8: 909–915. 19221498 10.4161/cc.8.6.7933

[12] Guba M, von Breitenbuch P, Steinbauer M, Koehl G, Flegel S, et al (2002) Rapamycin inhibits primary and metastatic tumor growth by antiangiogenesis: involvement of vascular endothelial growth factor. Nat Med 8: 128–135. 11821896 10.1038/nm0202-128

[13] Hidalgo M, Rowinsky EK (2000) The rapamycin-sensitive signal transduction pathway as a target for cancer therapy. Oncogene 19: 6680–6686. 11426655 10.1038/sj.onc.1204091

[14] Uhrbom L, Nerio E, Holland EC (2004) Dissecting tumor maintenance requirements using bioluminescence imaging of cell proliferation in a mouse glioma model. Nature Med 10: 1257–1260. 15502845 10.1038/nm1120

[15] Frost P, Moatamed F, Hoang B, Shi Y, Gera J, et al (2004) In vivo antitumor effects of the mTOR inhibitor CCI-779 against human multiple myeloma cells in a xenograft model. Blood 104: 4181–4187. 15304393 10.1182/blood-2004-03-1153

[16] Xing D, Orsulic S (2005) A genetically defined mouse ovarian carcinoma model for the molecular characterization of pathway-targeted therapy and tumor resistance. Proc Natl Acad Sci U S A 102: 6936–6941. 15860581 10.1073/pnas.0502256102 PMC1087513

[17] Werner H, Bruchim I (2009) The insulin-like growth factor-I receptor as an oncogene. Arch Physiol Biochem 115: 58–71. 19485702 10.1080/13813450902783106

[18] Bruchim I, Attias Z, Werner H (2009) Targeting the IGF1 axis in cancer proliferation. Expert Opin Ther Targets 13: 1179–1192. 19663648 10.1517/14728220903201702

[19] Cantrell LA, Zhou C, Mendivil A, Malloy KM, Gehrig PA, et al (2010) Metformin is a potent inhibitor of endometrial cancer cell proliferation–implications for a novel treatment strategy. Gynecol Oncol 116: 92–98. 19822355 10.1016/j.ygyno.2009.09.024 PMC2789879

[20] Xie Y, Wang YL, Yu L, Hu Q, Ji L, et al (2011) Metformin promotes progesterone receptor expression via inhibition of mammalian target of rapamycin (mTOR) in endometrial cancer cells. J Steroid Biochem Mol Biol 126: 113–120. 21168492 10.1016/j.jsbmb.2010.12.006

[21] Werner H (2009) For debate: the pathophysiological significance of IGF-I receptor overexpression: new insights. Pediatr Endocrinol Rev 7: 2–5. 19696710

[22] Bruchim I, Attias Z, Werner H (2009) Targeting the IGF1 axis in cancer proliferation. Expert Opin Ther Targets 13: 1179–1192. 19663648 10.1517/14728220903201702

[23] Pollak M (2008) Insulin and insulin-like growth factor signalling in neoplasia. Nature Rev Cancer 8: 915–928. 19029956 10.1038/nrc2536

[24] Werner H (2012) Tumor suppressors govern insulin-like growth factor signaling pathways: implications in metabolism and cancer. Oncogene 31: 2703–2714. 21963847 10.1038/onc.2011.447

[25] Gunter MJ, Hoover DR, Yu H, Wassertheil-Smoller S, Manson JE, et al (2008) A prospective evaluation of insulin and insulin-like growth factor-I as risk factors for endometrial cancer. Cancer Epidemiol Biomarkers Prev 17: 921–929. 18398032 10.1158/1055-9965.EPI-07-2686 PMC3090086

[26] Augustin LS, Polesel J, Bosetti C, Kendall CW, La Vecchia C, et al (2003) Dietary glycemic index, glycemic load and ovarian cancer risk: a case-control study in Italy. Ann Oncol 14: 78–84. 12488297 10.1093/annonc/dkg022

[27] Zhou G, Myers R, Li Y, Chen Y, Shen X, et al (2001) Role of AMP-activated protein kinase in mechanism of metformin action. J Clin Invest 108: 1167–1174. 11602624 10.1172/JCI13505 PMC209533

[28] Sarfstein R, Bruchim I, Fishman A, Werner H (2011) The mechanism of action of the histone deacetylase inhibitor vorinostat involves interaction with the insulin-like growth factor signaling pathway. PLoS One 6: e24468. 21931726 10.1371/journal.pone.0024468 PMC3169604

[29] Sarfstein R, Belfiore A, Werner H (2010) Identification of Insulin-Like Growth Factor-I Receptor (IGF-IR) Gene Promoter-Binding Proteins in Estrogen Receptor (ER)-Positive and ER-Depleted Breast Cancer Cells. Cancers 2: 233–261. 24281069 10.3390/cancers2020233 PMC3835077

[30] Seino S, Seino M, Nishi S, Bell GI (1989) Structure of the human insulin receptor gene and characterization of its promoter. Proc Natl Acad Sci U S A 86: 114–118. 2911561 10.1073/pnas.86.1.114 PMC286414

[31] Vindelov LL, Christensen IJ, Nissen NI (1983) A Detergent-Trypsin Method for the Preparation of Nuclei for Flow Cytometric DNA Analysis. Cytometry 3: 323–327. 6188586 10.1002/cyto.990030503

[32] Bhaskar PT, Hay N (2007) The two TORCs and Akt. Dev Cell 12: 487–502. 17419990 10.1016/j.devcel.2007.03.020

[33] Guertin DA, Sabatini DM (2007) Defining the role of mTOR in cancer. Cancer Cell 12: 9–22. 17613433 10.1016/j.ccr.2007.05.008

[34] Zakikhani M, Blouin MJ, Piura E, Pollak MN (2006) Metformin and rapamycin have distinct effects on the AKT pathway and proliferation in breast cancer cells. Breast Cancer Res Treat 123: 271–279. 10.1007/s10549-010-0763-9 20135346

[35] Sarfstein R, Maor S, Reizner N, Abramovitch S, Werner H (2006) Transcriptional regulation of the insulin-like growth factor-1 receptor in breast cancer. Mol Cell Endocrinol 252: 241–246. 16647191 10.1016/j.mce.2006.03.018

[36] Kiang JG, Bowman PD, Wu BW, Hampton N, Kiang AG, et al (2004) Geldanamycin treatment inhibits hemorrhage-induced increases in KLF6 and iNOS expression in unresuscitated mouse organs: role of inducible HSP70. J Appl Physiol 97: 564–569. 15090481 10.1152/japplphysiol.00194.2004

[37] Choi SH, Kim YW, Kim SG (2010) AMPK-mediated GSK3beta inhibition by isoliquiritigenin contributes to protecting mitochondria against iron-catalyzed oxidative stress. Biochem Pharmacol 79: 1352–1362. 20026081 10.1016/j.bcp.2009.12.011

[38] Ciaraldi TP, Oh DK, Christiansen L, Nikoulina SE, Kong AP, et al (2006) Tissue-specific expression and regulation of GSK-3 in human skeletal muscle and adipose tissue. Am J Physiol Endocrinol Metab 291: E891–898. 16757548 10.1152/ajpendo.00176.2006

[39] Song J, Ren P, Zhang L, Wang XL, Chen L, et al (2010) Metformin reduces lipid accumulation in macrophages by inhibiting FOXO1-mediated transcription of fatty acid-binding protein 4. Biochem Bioph Res Commun 393: 89–94. 10.1016/j.bbrc.2010.01.086 20102700

[40] Libby G, Donnelly LA, Donnan PT, Alessi DR, Morris AD, et al (2009) New users of metformin are at low risk of incident cancer: a cohort study among people with type 2 diabetes. Diabetes Care 32: 1620–1625. 19564453 10.2337/dc08-2175 PMC2732153

[41] Currie CJ, Poole CD, Gale EA (2009) The influence of glucose-lowering therapies on cancer risk in type 2 diabetes. Diabetologia 52: 1766–1777. 19572116 10.1007/s00125-009-1440-6

[42] Jiralerspong S, Palla SL, Giordano SH, Meric-Bernstam F, Liedtke C, et al (2009) Metformin and pathologic complete responses to neoadjuvant chemotherapy in diabetic patients with breast cancer. J Clin Oncol 27: 3297–3302. 19487376 10.1200/JCO.2009.19.6410 PMC2736070

[43] Dong L, Zhou Q, Zhang Z, Zhu Y, Duan T, et al (2012) Metformin sensitizes endometrial cancer cells to chemotherapy by repressing glyoxalase I expression. J Obstet Gynaecol Res 38: 1077–1085. 22540333 10.1111/j.1447-0756.2011.01839.x

[44] Hanna RK, Zhou C, Malloy KM, Sun L, Zhong Y, et al (2012) Metformin potentiates the effects of paclitaxel in endometrial cancer cells through inhibition of cell proliferation and modulation of the mTOR pathway. Gynecol Oncol 125: 458–469. 22252099 10.1016/j.ygyno.2012.01.009 PMC3322276

[45] Hollstein M, Sidransky D, Vogelstein B, Harris CC (1991) p53 mutations in human cancers. Science 253: 49–53. 1905840 10.1126/science.1905840

[46] Jin X, Gossett DR, Wang S, Yang D, Cao Y, et al (2004) Inhibition of AKT survival pathway by a small molecule inhibitor in human endometrial cancer cells. Br J Cancer 91: 1808–1812. 15505622 10.1038/sj.bjc.6602214 PMC2410058

[47] Reynolds RK, Hu C, Baker VV (1998) Transforming growth factor-alpha and insulin-like growth factor-I, but not epidermal growth factor, elicit autocrine stimulation of mitogenesis in endometrial cancer cell lines. Gynecol Oncol 70: 202–209. 9740691 10.1006/gyno.1998.5089

[48] Kang-Park S, Lee YI, Lee YI (2003) PTEN modulates insulin-like growth factor II (IGF-II)-mediated signaling; the protein phosphatase activity of PTEN downregulates IGF-II expression in hepatoma cells. FEBS Lett 545: 203–208. 12804776 10.1016/s0014-5793(03)00535-0

[49] Zhao H, Dupont J, Yakar S, Karas M, LeRoith D (2004) PTEN inhibits cell proliferation and induces apoptosis by downregulating cell surface IGF-IR expression in prostate cancer cells. Oncogene 23: 786–794. 14737113 10.1038/sj.onc.1207162

[50] Korch C, Spillman MA, Jackson TA, Jacobsen BM, Murphy SK, et al (2012) DNA profiling analysis of endometrial and ovarian cell lines reveals misidentification, redundancy and contamination. Gynecol Oncol 127: 241–248. 22710073 10.1016/j.ygyno.2012.06.017 PMC3432677

[51] Santin AD, Zhan F, Cane S, Bellone S, Palmieri M, et al (2005) Gene expression fingerprint of uterine serous papillary carcinoma: identification of novel molecular markers for uterine serous cancer diagnosis and therapy. Br J Cancer 92: 1561–1573. 15785748 10.1038/sj.bjc.6602480 PMC2362016

[52] Attias-Geva Z, Bentov I, Ludwig DL, Fishman A, Bruchim I, et al (2011) Insulin-like growth factor-I receptor (IGF-IR) targeting with monoclonal antibody cixutumumab (IMC-A12) inhibits IGF-I action in endometrial cancer cells. Eur J Cancer 47: 1717–1726. 21450456 10.1016/j.ejca.2011.02.019

[53] Zhuang Y, Miskimins WK (2008) Cell cycle arrest in Metformin treated breast cancer cells involves activation of AMPK, downregulation of cyclin D1, and requires p27Kip1 or p21Cip1. J Mol Signal 3: 18. 19046439 10.1186/1750-2187-3-18 PMC2613390

[54] Bost F, Sahra IB, Le Marchand-Brustel Y, Tanti J-F (2012) Metformin and cancer therapy. Curr Opin Oncol 24: 103–108. 22123231 10.1097/CCO.0b013e32834d8155

[55] Vivanco I, Sawyers CL (2002) The phosphatidylinositol 3-Kinase AKT pathway in human cancer. Nat Rev Cancer 2: 489–501. 12094235 10.1038/nrc839

[56] Bjornsti MA, Houghton PJ (2004) The TOR pathway: a target for cancer therapy. Nat Rev Cancer 4: 335–348. 15122205 10.1038/nrc1362

[57] Oldham S, Hafen E (2003) Insulin/IGF and target of rapamycin signaling: a TOR de force in growth control. Trends Cell Biol 13: 79–85. 12559758 10.1016/s0962-8924(02)00042-9

[58] Beurel E, Kornprobst M, Blivet-Van Eggelpoel MJ, Ruiz-Ruiz C, Cadoret A, et al (2004) GSK-3beta inhibition by lithium confers resistance to chemotherapy-induced apoptosis through the repression of CD95 (Fas/APO-1) expression. Exp Cell Res 300: 354–364. 15475000 10.1016/j.yexcr.2004.08.001

[59] Liu S, Yu S, Hasegawa Y, Lapushin R, Xu HJ, et al (2004) Glycogen synthase kinase 3beta is a negative regulator of growth factor-induced activation of the c-Jun N-terminal kinase. J Biol Chem 279: 51075–51081. 15466414 10.1074/jbc.M408607200 PMC5328675

